# Highly sensitized kidney transplant candidates: integrating acceptable mismatch, desensitization, imlifidase, and emerging immune-cell targeting strategies

**DOI:** 10.3389/fimmu.2026.1868642

**Published:** 2026-06-30

**Authors:** Stefan Reuter, Nils Lachmann, Hans de Ferrante, Georg A. Böhmig, Klemens Budde

**Affiliations:** 1 Department of Internal Medicine D, Transplant Nephrology, University of Muenster, Muenster, Germany; 2 Institute of Transfusion Medicine, Tissue Typing/Human Leukocyte Antigen (HLA), Charité – Universitätsmedizin Berlin, Berlin, Germany; 3 Eurotransplant International Foundation, Leiden, Netherlands; 4 Department of Medicine III, Medical University of Vienna, Vienna, Austria; 5 Department of Nephrology and Medical Intensive Care, Charité – Universitätsmedizin Berlin, Berlin, Germany

**Keywords:** acceptable mismatch, BCMA, CAR-T cells, desensitization, donor frequency, highly sensitized, imlifidase, kidney transplantation

## Abstract

Highly sensitized kidney transplant candidates are difficult to transplant because anti-human leukocyte antigen (HLA) sensitization restricts access to immunologically compatible organs, as transplantation across donor-specific antibodies (DSA) increases the risk of antibody-mediated rejection (AMR) and premature graft loss. Management should therefore move beyond antibody characteristics alone and assess whether a compatible offer remains realistic for the individual patient within the relevant allocation system. This review proposes a sequential, compatibility-first access framework. Compatible transplantation should remain the preferred goal and may be achieved through kidney paired exchange, compatible living-donor pathways, sensitization-aware prioritization, and dedicated allocation programs. International experience shows that allocation design can mitigate access restrictions for moderately sensitized candidates, while highly sensitized candidates require more targeted prioritization. The Eurotransplant acceptable mismatch program exemplifies this principle by combining expert-defined acceptable antigens with allocation priority, thereby promoting compatible transplantation with excellent outcomes in a restricted highly sensitized cohort without therapeutic crossing of the immunological barrier. Compatible pathways should be tried and optimized before intervention-based strategies are considered. In candidates without a realistic compatible option within a reasonable timeframe, conventional desensitization, risk-adapted delisting of selected unacceptable antigens, or imlifidase-enabled transplantation may become justified, particularly when severe dialysis-related problems exist. Imlifidase can rapidly enable selected positive-crossmatch deceased-donor transplantation, but AMR remains frequent and strict governance, candidate selection, and posttransplant surveillance are mandatory. Novel AMR treatments, including CD38-directed therapies, may improve the long-term feasibility of barrier-crossing strategies. Emerging B-cell and plasma-cell targeting approaches, including interleukin-6-, CAR-T-cell-, and BCMA-directed strategies, remain investigational. Optimal care requires integrating immunologic precision, allocation design, intervention thresholds, and structured monitoring into an individualized compatibility-first strategy.

## 1 Introduction

Highly sensitized kidney transplant candidates remain among the most difficult patients to transplant, not because a single therapeutic solution is missing, but because successful access depends on a combination of complementary strategies. Sensitization limits access to immunologically compatible donor organs long before it becomes a peri-transplant immunological problem (1). Management therefore depends on the interaction between antibody characteristics, unacceptable-antigen assignment, expected waiting time, dialysis-associated morbidity, urgency, donor-pool composition, and access-expanding options such as kidney paired donation (KPD), also referred to as kidney paired exchange, acceptable mismatch (AM) allocation, or desensitization (2, 3).

The preferred goal remains timely transplantation with a compatible, virtual and physical crossmatch-negative donor without the need for additional incompatibility-directed therapy beyond standard immunosuppression (4, 5). Accordingly, sensitization should primarily be conceptualized as an access problem rather than a desensitization problem. This distinction is important because compatible allocation and intervention-based access are not equivalent pathways.

In the AM program, experts define an extended HLA phenotype for each candidate by combining self HLA antigens with acceptable antigens, usually antigens against which no HLA antibodies have ever been detected after detailed antibody history and epitope-pattern analysis. Access to compatible transplantation is then facilitated by giving allocation priority for donor kidneys carrying only self or acceptable HLA antigens. Thus, the success of the AM program depends on the combination of expert-defined immunologic safety and allocation priority, rather than on the definition of acceptable antigens alone. This strategy has translated into high offer and transplant rates, with organ offers made for roughly 80% of AM candidates and more than 60% of candidates transplanted within 3 years after AM listing, together with excellent long-term outcomes (5). Outcome data from the AM program support the durability and immunological safety of this approach (6–8). A Europe-wide simulation further suggests that broader AM collaboration across international allocation schemes could improve access for selected long-waiting highly sensitized patients (9).

Desensitization should be viewed as an access-enabling intervention for selected candidates in whom allocation-based strategies alone are unlikely to succeed in a given timeframe, especially for patients with urgent transplant need due to dialysis-associated problems (2). Imlifidase occupies a more specific role as a rapid access-enabling strategy in highly selected deceased-donor settings (10). Its clinical implementation is associated with frequent AMR and therefore requires governed selection and surveillance pathways (11–13). Emerging immune-cell-targeting strategies, including chimeric antigen receptor (CAR)-T approaches and B-cell maturation antigen (BCMA)-directed immune engagers, remain investigational (14–17).

**Figure 1:**
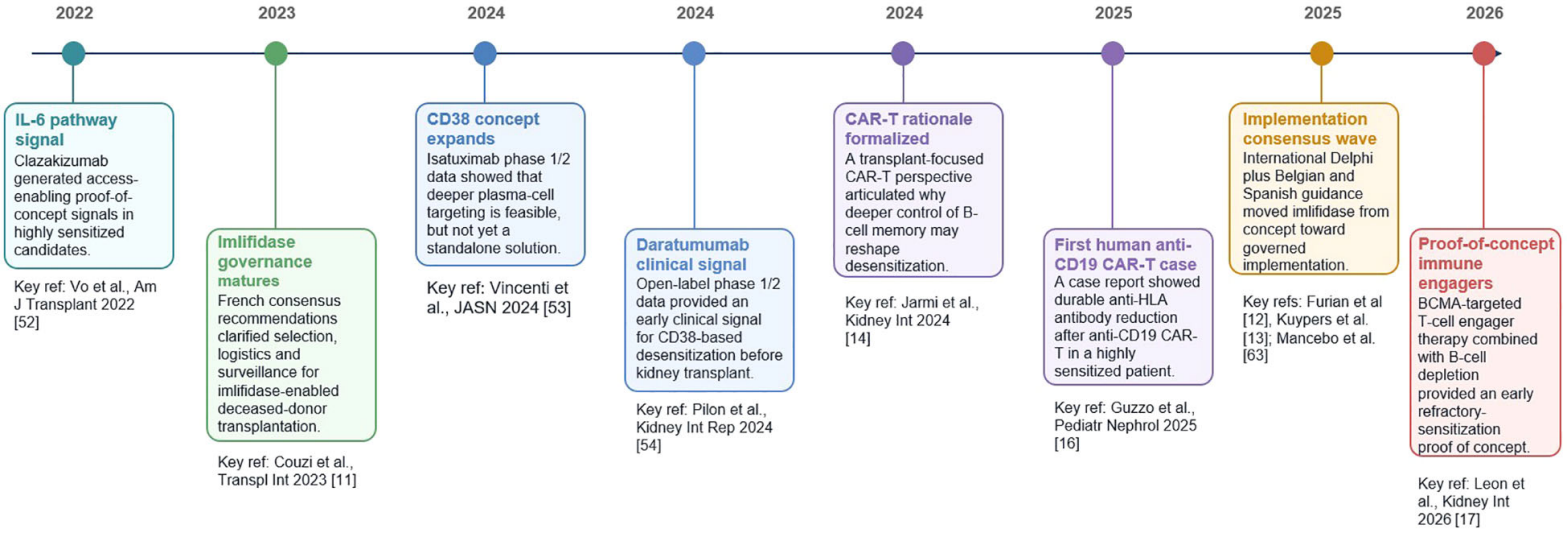
Timeline of access-enabling and immune-cell-targeting strategies for highly sensitized kidney transplant candidates. The horizontal arrow denotes time; boxes mark evidence milestones and implementation papers relevant to the field.

**Figure 2:**
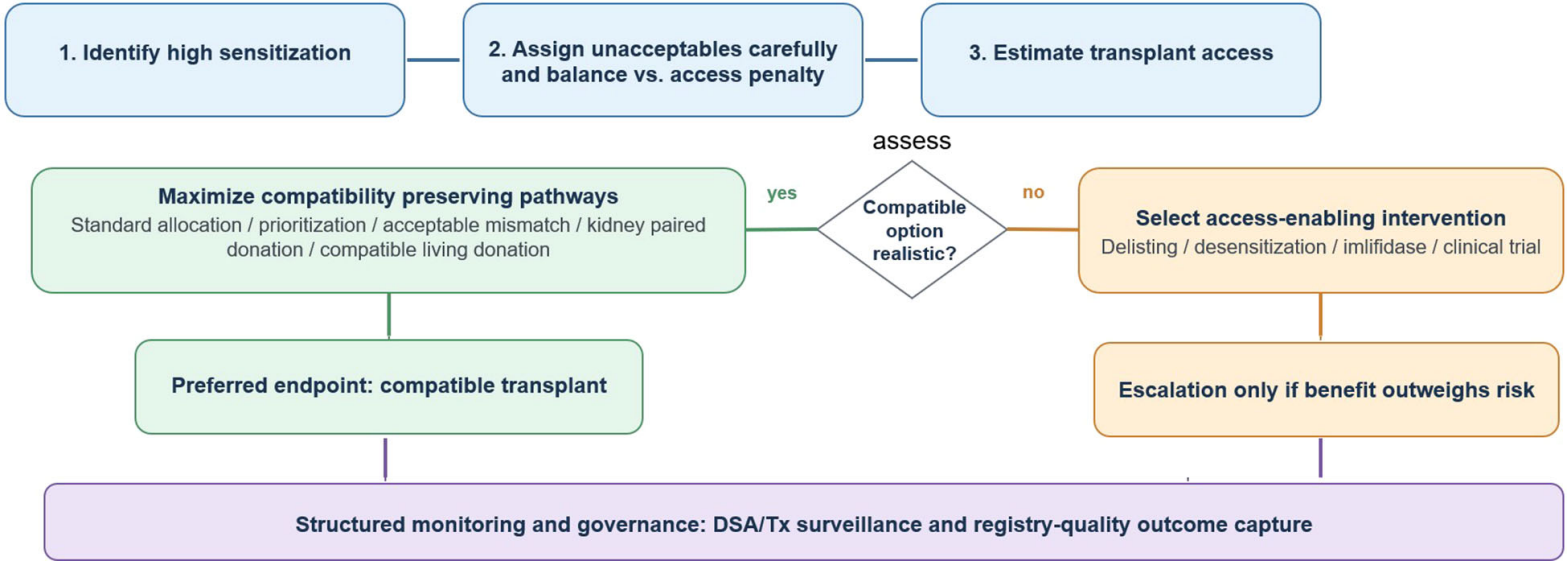
Practical framework for the management of highly sensitized kidney transplant candidates. Compatible transplantation remains the preferred endpoint; intervention-based strategies are reserved for the subgroup without a realistic compatible option.

This review proposes a sequential, compatibility-first access framework for the management of sensitized patients that begins with immunological characterization of the candidate, proceeds to transplantability assessment, and then considers when intervention may be justified. **Figure 1** places the major access-enabling and immune-cell-targeting milestones on an implementation timeline, **Figure 2** translates the compatibility-first concept into a practical clinical pathway, and the core take-home messages are summarized in **Table 1**.

**Table 1:** Core take-home messages of the compatibility-first framework.

Message	Practical implication
Sensitization is primarily an access problem.	Management should first estimate whether a compatible donor offer remains realistic within the relevant allocation system.
Compatible transplantation remains preferred whenever achievable.	Allocation, AM programs, KPD, and compatible living-donor routes should be optimized before crossing an HLA barrier.
Allocation design modifies the access penalty of HLA sensitization.	The same cPRA, vPRA, or cRF can have different clinical meanings across allocation systems and donor pools.
Desensitization benefit is context-dependent.	Risk-benefit assessment must include local allocation alternatives, dialysis risk, comorbidity, immunologic risk, and resource capacity.
Escalation is sequential, not binary.	Intervention-based strategies should be reserved for candidates without a realistic compatible option or with clinical urgency that makes further waiting unsafe.

## 2 Defining the highly sensitized kidney transplant candidate

There is no single universally sufficient definition of a highly sensitized kidney transplant candidate. Historically, a panel-reactive antibody (PRA) level of at least 85%, assessed by complement-dependent cytotoxicity (CDC) against a cell panel, was used to define highly sensitized patients, based on the observation that such patients accumulated on the waiting list (5, 18). With the introduction of solid-phase assays (SPA), sensitization is now more commonly expressed as calculated panel reactive antibody (cPRA), virtual PRA (vPRA), or calculated reaction frequency (cRF) (19). These metrics all estimate the proportion of a defined donor population carrying antigens listed as unacceptable, but each does so against a reference donor population and with allocation-system-specific assumptions.

A benefit of calculated sensitization metrics is that they are more directly linked to donor-pool restriction than historical PRA. Their major limitation is that they depend on how antigens are classified as unacceptable. CDC-reactive antibodies generally leave little room for interpretation and are usually considered unacceptable. By contrast, there is no universally standardized protocol for translating SPA reactivity into unacceptable-antigen assignment. The mere identification of a potential donor-specific antibody (DSA) by solid-phase testing does not, by itself, define the immunological risk of proceeding with transplantation across that antibody. the European Society for Organ Transplantation (ESOT) has emphasized that antibody plausibility, previous immunizing events, donor-specific antibody status, crossmatch results, HLA compatibility, and immunologic history should be integrated and weighed against the access restriction caused by listing antigens as unacceptable (4, 20, 21).

To what degree listing unacceptables reduces transplant access is allocation-system- and context-dependent. In Eurotransplant, immunized candidates face reduced access compared with non-immunized candidates in regular Eurotransplant Kidney Allocation System (ETKAS) allocation, with transplantation rates declining rapidly above a vPRA of 85% (22). German data similarly show that donor-pool restriction by unacceptable antigens prolongs waiting time and reduces transplant probability, most clearly above vPRA of 85% and dramatically above vPRA of 95% (23). By contrast, recent French data suggest that candidates with calculated sensitization levels between 85% and 95% are not disadvantaged compared with non-immunized candidates, while access declines sharply at higher levels (24). Also within allocation systems, the same calculated sensitization level may translate into different access probabilities depending on ABO blood group and HLA match requirements. Key definitions and determinants of sensitization and transplantability are summarized in **Table 2**.

**Table 2:** Definitions and determinants of sensitization and transplantability.

Parameter	What it reflects	Clinical relevance	Main limitations
PRA	Proportion of a cell panel reacting with recipient serum, historically assessed by complement-dependent cytotoxicity	Historical measure of broad HLA sensitization; a PRA threshold of ≥85% was traditionally used to identify candidates accumulating on the waiting list	Panel- and center-dependent; not directly comparable with cPRA, vPRA, or cRF; does not fully reflect donor-population-specific transplantability
cPRA/vPRA/cRF	Estimated proportion of a defined donor population expected to be incompatible based on listed unacceptable antigens	Useful summary measure of sensitization-related donor restriction and strongly associated with transplant access	Dependent on unacceptable-antigen assignment; does not by itself capture ABO blood group, HLA match quality, allocation priority, or waiting-list competition
Donor-specific antibodies (DSA)	Antibodies directed against donor HLA	Central determinant of positive crossmatch risk and antibody-mediated rejection risk	Clinical impact depends on specificity, strength, complement-binding characteristics, temporal pattern, and clinical context
Virtual crossmatch	Predicted donor–recipient compatibility based on donor HLA typing and recipient antibody profile	Enables rapid allocation decisions and pretransplant immunologic risk stratification	Accuracy depends on correct antibody interpretation, complete donor typing, and appropriate unacceptable-antigen assignment
Physical crossmatch	Functional test of donor–recipient immunologic compatibility	Important confirmatory tool, especially in complex or high-risk settings	Time-sensitive, method-dependent, and not always available in urgent allocation settings
Unacceptable antigens	Donor antigens judged clinically unsafe for a given recipient	Core determinant of transplantability; directly influences donor eligibility and allocation access	Overassignment may unnecessarily restrict access; underassignment may increase immunologic risk
Immunizing history	Source and expected magnitude or complexity of sensitization, including prior transplantation, pregnancy, and transfusion	Informs the plausibility and clinical relevance of detected antibodies and should guide unacceptable-antigen assignment	Labor-intensive to obtain and interpret; documentation may be incomplete; not consistently integrated into antibody assessment and unacceptable-antigen assignment
ABO blood group	ABO-related donor availability constraint	Can materially affect access even at similar levels of HLA sensitization	Often overlooked when sensitization is discussed only in HLA-centered terms
Donor frequency	Proportion of donors in a reference donor population who are ABO-compatible or ABO-identical and do not carry unacceptable antigens for a given candidate	More access-oriented than cPRA, vPRA, or cRF because it incorporates blood group and unacceptable antigens	Does not account for HLA match quality or competition from other waiting-list candidates
Favorably matched donor frequency	Proportion of donors who are compatible and also meet a predefined HLA match-quality threshold	Quantifies how common immunologically favorable donors are for a candidate	Still donor-centered; does not fully capture whether the candidate is likely to receive the offer under the used allocation rules and waiting-list competition
Matchability	Probability that a candidate will receive a favorable offer, accounting for donor phenotype distribution and competition from other candidates	Allocation-aware measure of real-world access; particularly relevant for rare HLA phenotypes and homozygosity	Requires allocation-system-specific modelling and is not routinely available in all systems.
Waiting time/access trajectory	Longitudinal evidence of whether compatible transplantation remains realistic	Helps distinguish a difficult-to-transplant candidate from a true no-realistic-compatible-option candidate	Requires interpretation within the relevant allocation environment and prioritization framework

PRA, panel reactive antibody; cPRA, calculated panel reactive antibody; vPRA, virtual panel reactive antibody; cRF, calculated reaction frequency; DSA, donor-specific antibody. Calculated sensitization metrics are clinically useful but insufficient in isolation. In highly sensitized kidney transplant candidates, transplantability is determined by the interaction between antibody profile, unacceptable-antigen assignment, crossmatch context, immunizing history, ABO blood group, donor frequency, HLA match quality, allocation priority, waiting-list competition, and observed access over time.

For the purpose of this review, and in line with ESOT recommendations, high sensitization identifies an at-risk population rather than a uniform clinical state. After immunological risk has been assessed, context matters for estimating real transplantability within the relevant allocation system. This assessment should integrate antibody breadth, unacceptable-antigen assignment, ABO blood group, donor frequency, HLA match requirements, waiting-list competition, allocation priority, and the candidate’s observed access trajectory, together with clinical urgency, comorbidities, current medical status, prior transplant and immunizing history, and patient preferences. Thus, the clinically relevant question is not only whether a candidate is highly sensitized, but whether compatible transplantation remains realistically achievable and clinically appropriate.

## 3 The preference for immunologically compatible transplantation

The first clinical management question is not which desensitization strategy to use, but whether compatible transplantation remains realistically achievable based on an individual risk-benefit assessment. For highly sensitized candidates, compatible transplantation should remain the preferred strategy because it avoids deliberate transplantation across a defined humoral barrier, usually permits standard immunosuppression without peri-transplant barrier-breaking therapy, and is associated with a substantially lower risk of early AMR than DSA-positive transplantation (4, 5).

Barrier-crossing strategies add treatment burden, infectious and toxic risks, costs, posttransplant surveillance, and uncertainty regarding long-term efficacy; premature graft loss may also return the patient to dialysis in an even more sensitized and medically vulnerable state.

This does not mean that HLA-incompatible transplantation is never justified. HLA-incompatible living-donor transplantation can provide a survival benefit over remaining on dialysis for selected patients after balancing risks and benefits (25). Such strategies may therefore be appropriate only when compatible access is very unlikely and the expected benefit of earlier transplantation outweighs the immunologic and treatment risks.

In this compatibility-first framework, compatible transplantation should be understood broadly. It may be achieved through standard allocation, dedicated prioritization schemes, AM allocation, KPD, or compatible living-donor pathways (3, 5). The main access strategies discussed in this review are summarized in **Table 3**.

**Table 3:** Access strategies for highly sensitized kidney transplant candidates.

Strategy	Main objective	Best suited setting	Major strengths	Main limitations	Current role
Standard compatible allocation and sensitization-aware prioritization	Increase access to a crossmatch-negative compatible donor within the routine allocation system	All sensitized candidates	Preserves the most favorable immunologic starting point; avoids treatment-related burden; can reduce the need for therapeutic barrier-breaking if allocation design is effective	Effectiveness depends on allocation rules, donor availability, blood group, HLA match requirements, and degree of prioritization	Foundational system-level strategy
Acceptable mismatch allocation	Provide priority access to kidneys carrying self or immunologically acceptable HLA antigens	Highly sensitized candidates within structured allocation systems	Expands compatible access without therapeutic barrier-breaking through the combination of expert-defined acceptable antigens and allocation priority; strong Eurotransplant outcome data	Requires expert HLA interpretation, robust unacceptable-antigen governance, and a sufficient donor pool; not sufficient for all low-frequency phenotypes	Core allocation-based access strategy
Kidney paired donation/exchange/compatible living-donor pathways	Circumvent incompatibility while preserving compatibility	Incompatible living-donor settings Also relevant for compatible pairs and chain-based strategies in larger registries.	May avoid desensitization entirely; biologically preferable when feasible Compatible pairs may extend chains and improve match quality; can be combined with limited low-risk desensitization or low-risk ABO-incompatible transplantation in selected cases.	Logistically complex; dependent on program size, donor availability, and matching probability Effectiveness is reduced in small pools and in candidates with cPRA/vPRA >95% or rare compatibility requirements.	Important adjunct compatible pathway Complementary to AM allocation rather than interchangeable with it.
Risk-adapted delisting of selected unacceptable antigens	Expand the virtual compatible donor space by reclassifying selected antibody specificities after structured risk assessment	Extremely sensitized candidates with very low donor frequency and prolonged absence of offers	Directly increases potential offer opportunities; can be combined with crossmatch safeguards and desensitization protocols	Risk of DSA rebound and antibody-mediated rejection if pathogenic antibodies are delisted; requires expert HLA interpretation and intensive monitoring	Emerging access-enabling strategy in specialized programs
Conventional desensitization	Reduce circulating anti-HLA antibodies sufficiently to permit transplantation across an HLA barrier	Mainly planned living-donor settings and selected controlled scenarios	Longest clinical experience; flexible protocol combinations; can be scheduled and monitored iteratively	Incomplete control of humoral memory; antibody rebound; infection risk; resource-intensive	Selective escalation strategy
Plasma-cell/IL-6–targeting pharmacologic approaches	Improve depth or durability of desensitization beyond conventional protocols	Selected highly sensitized candidates, mainly investigational or specialized-center use	Strong mechanistic rationale; may address limitations of standard antibody-lowering protocols	Clinical evidence remains limited, heterogeneous, and mostly nonstandardized	Emerging adjunctive strategy
Obinutuzumab-containing approaches	Achieve deeper B-cell depletion than standard anti-CD20 therapy	Selected highly sensitized candidates	Demonstrates potent pharmacodynamic B-cell depletion	Routine role in kidney-transplant desensitization remains uncertain	Investigational/adjunctive
Imlifidase	Rapidly convert an IgG-mediated positive crossmatch to enable transplantation	Highly selected deceased-donor settings with time-critical organ offers	Unique immediate access-enabling effect; creates a short transplantation window	DSA rebound and early antibody-mediated rejection risk; requires highly structured monitoring and experienced-center use High acquisition cost, time-critical logistics, early AMR risk around 40%, and uncertain generalizability of long-term benefit limit broad routine use.	Selective rescue/bridge strategy
CAR-T-cell approaches	Target deeper B-cell and humoral-memory compartments	Extremely selected rescue settings or clinical trials	Strong conceptual rationale for durable immune reset	Very limited transplant experience; major safety, feasibility, cost, and access questions remain	Early next-wave approach
Bispecific immune-engaging/BCMA-directed strategies	Target plasma-cell biology in refractory sensitization	Highly selected proof-of-concept settings	Mechanistically attractive for refractory humoral sensitization	Very early clinical evidence; durability, infectious risk, and implementation pathways unresolved	Investigational next-wave approach

AMR, antibody-mediated rejection; BCMA, B-cell maturation antigen; CAR-T, chimeric antigen receptor T cell; DSA, donor-specific antibody. Current evidence supports a compatible-first strategy. Acceptable mismatch allocation expands access through the combination of expert-defined acceptable antigens and allocation priority for compatible offers; the definition of acceptable antigens alone does not increase access. Intervention-based strategies should be reserved for candidates with no realistic compatible option after compatible allocation pathways have been exhausted.

## 4 Concepts for quantifying transplant access

Although cPRA, vPRA, and cRF are strongly associated with transplant access, they are not ideal stand-alone tools for estimating whether a candidate can realistically expect a compatible kidney offer. These metrics quantify expected donor incompatibility based on listed unacceptable antigens, but they do not account for all factors that shape real-world transplant access, including ABO blood group, HLA match quality requirements, allocation priority, or competition from other waitlisted candidates (19).

A more access-oriented parameter is donor frequency. In Eurotransplant, donor frequency is defined as the proportion of donors in a reference donor population who do not carry the unacceptable antigens for a given candidate and are either ABO-identical or ABO-compatible (18). This addresses one limitation of cPRA, vPRA, and cRF by incorporating ABO blood group into the estimate of compatible donor availability. Donor frequency therefore helps translate antibody-defined incompatibility into an allocation-relevant estimate of how frequently a potentially suitable donor exists.

In allocation systems in which HLA match quality is emphasized, transplant access is better captured by the proportion of donors who are compatible and meet a predefined HLA match-quality threshold. We refer to this as favorably matched donor frequency. In certain regions, favorably-matched donor frequency is already used for allocation. For example, in France, allocation points are awarded for the number of blood-group identical, compatible donors with at most 3 HLA mismatches (24), while in the United Kingdom, points are awarded for the number of compatible donors with a favorable HLA-B and HLA-DR match (29).

Donor frequency-based metrics indicate whether compatible, or favorably matched compatible, donors exist within a reference donor population. However, real-world access also depends on allocation rules and competition among candidates for the same donor organs. Matchability, as proposed by Gilks and colleagues, addresses this limitation by estimating the candidate-specific probability of receiving a favorable or well-matched offer (28). A candidate may have an apparently acceptable donor frequency but poor matchability if the same favorable donors are in high demand. Conversely, lower donor frequency does not necessarily imply poorer access if competition for those donors is limited. Recent registry data using a matchability score also show that difficult-to-match profiles are associated with reduced deceased-donor transplant access and inferior posttransplant outcomes in Eurotransplant (30).

This distinction is relevant for candidates with rare blood groups, such as B or AB, who may compete within smaller blood group-identical waiting lists, and for homozygous candidates, who face substantial competition from heterozygous candidates for the same well-matched donors. Eurotransplant analyses illustrate that rare HLA phenotypes and homozygosity can both reduce the probability of receiving a favorable offer despite apparently acceptable donor frequency. Matchability may therefore provide a more allocation-aware measure of real-world access than donor frequency alone.

Assessment of transplant access should therefore move from antibody breadth alone to a layered approach: cPRA, vPRA, or cRF to estimate HLA-antibody-related incompatibility burden; donor frequency to estimate HLA- and ABO-compatible donor availability; favorably matched donor frequency to incorporate match quality; and matchability to approximate the chance of a favorable offer under allocation-system-specific competition. These concepts help distinguish candidates who are difficult to transplant from those with no realistic compatible option.

## 5 Immunizing events and their clinical relevance

Clinically meaningful HLA sensitization most commonly follows prior transplantation, pregnancy, or blood transfusion (31, 32). These events differ in immunobiology and clinical consequence. Prior transplantation is often associated with broad, deep, and persistent alloimmune memory, whereas pregnancy, particularly repeated pregnancies, may generate individually variable complex antibody responses that remain clinically relevant long after the sensitizing event (33, 34). Repeated or substantial transfusion exposure can also contribute meaningfully to sensitization, especially to HLA class I, although its effect varies with exposure intensity and composition of the transfused product (31, 32).

The careful assessment of a patient’s immunizing history must therefore be integrated into pre-transplant risk stratification from the outset. Candidates with multiple immunizing events and broad HLA class I and class II reactivity are particularly likely to require highly individualized and complex unacceptable-antigen assignment (4, 20). Whenever available, knowledge of the HLA type of the sensitizing source, such as a previous donor or the biological father of a child, can be highly informative for interpreting antibody plausibility, repeated mismatches, and historical or fluctuating reactivities. Additional HLA typing of relevant sensitizing sources may therefore be considered when feasible, particularly if it helps distinguish biologically plausible alloantibody reactivity from uncertain or potentially overcalled assay signals. This approach supports a more accountable assignment of unacceptable antigens and helps balance immunologic safety against the access penalty created by donor exclusion.

From a mechanistic standpoint, immunizing events are not interchangeable stimuli that induce identical immune memory. Pregnancy-related sensitization should not be underestimated, because clinically relevant alloimmune memory may persist even when individual assay signals fluctuate over time (34). These qualitative aspects of sensitization are directly relevant to unacceptable-antigen assignment and to later decisions about delisting or escalation.

## 6 Antibody characterization: beyond MFI alone

The central laboratory challenge in highly sensitized candidates is not the detection of anti-HLA antibodies per se, but the estimation of their clinical relevance and, consequently, the discrimination between acceptable and unacceptable HLA mismatches. Modern solid-phase assays (SPA) have substantially increased the specificity and sensitivity of anti-HLA antibody detection (35, 36). However, this increased sensitivity has also increased interpretive complexity. Mean fluorescence intensity (MFI), the readout of the most commonly used Luminex-based SPA, is insufficient as a stand-alone decision metric because it is a semiquantitative assay signal rather than a direct measure of immunological risk (35–37).

Interpretation must remain contextual and integrate reproducibility, serum dilution behavior, plausibility based on sensitizing events, reactivity pattern, antigen specificity and epitopes, and concordance with virtual or physical crossmatch findings. Standardization initiatives such as the Sensitization in Transplantation: Assessment of Risk (STAR) working group and earlier consensus recommendations have raised awareness, improved terminology, and supported risk framing, but they do not remove the need for expert integration (37, 38).

The field is also moving beyond standard IgG detection. Complement-binding capacity, antibody titers by serum dilution, and IgG subclass profiling may add biological information in selected cases, but current evidence does not support their isolated use for defining unacceptable antigens or transplant eligibility of highly sensitized candidates in daily routine (39–41). This is particularly important in candidates with dramatic donor restriction, because unwarranted overassignment of unacceptable antigens may convert a difficult-to-transplant candidate into one who is practically untransplantable.

Flow cytometric crossmatch (FCXM) testing may provide additional functional information, particularly when SPA results are difficult to interpret or when low-level donor-specific reactivity is suspected. However, like complement-binding assays and IgG subclass testing, FCXM results should be interpreted in context rather than used as an isolated determinant of unacceptable-antigen assignment.

## 7 Improving access through allocation design and priority

Allocation design can substantially modify the access penalty associated with HLA sensitization. The same calculated sensitization level may translate into different transplant probabilities depending on how a given allocation system incorporates unacceptable antigens, ABO blood group, HLA match quality, waiting time, and prioritization. Thus, sensitization-related access barriers are not fixed biological facts alone, but are also shaped by policy choices within the allocation algorithm.

International experience illustrates this principle. In the United States, points are awarded based on the cPRA, which has improved access for many highly sensitized candidates, although candidates at the extreme end of sensitization remain difficult to transplant (3, 26). In France, points are awarded based on the taux de greffons incompatibles (TGI), which appears to preserve deceased-donor transplant access for candidates in the 85%-95% sensitization range, whereas access declines more sharply at higher levels (24). The United Kingdom has also incorporated donor compatibility and match-quality concepts into allocation, illustrating that standard allocation algorithms can be adapted to reduce access barriers for immunized or difficult-to-match candidates (29). These examples show that allocation reform can mitigate the disadvantage associated with sensitization, particularly for moderately to highly sensitized candidates.

For candidates with very high sensitization and low probability of transplantation in regular allocation, dedicated priority programs become particularly important. The Eurotransplant AM program is the most established example of such a strategy. Its distinctive feature, detailed above, is the operational coupling of expert-defined immunologic acceptability with allocation priority, enabling compatible transplantation without therapeutic crossing of an HLA barrier (5, 18).

The clinical success of this approach is well documented. Early reports showed that AM-based allocation could shorten waiting time while maintaining excellent graft outcomes (42). Subsequent Eurotransplant analyses showed superior graft survival with allocation based on proven acceptable antigens and low rejection rates comparable to those observed in nonsensitized recipients (6, 7). In the cohort reported by Heidt et al., organ offers were made for roughly 80% of AM candidates and more than 60% were transplanted within 3 years after AM listing (5). More recent long-term data further support the durability of AM-based allocation (8). A Europe-wide AM simulation suggests that broader cross-border collaboration may further improve access for selected long-waiting highly sensitized candidates (9).

Nevertheless, allocation-based strategies are not universally sufficient. Standard allocation reforms may reduce the disadvantage of moderate sensitization, and AM allocation may provide excellent access for many highly sensitized candidates, but patients with extremely low donor frequency, rare HLA phenotypes, broad antibody repertoires, or poor matchability may remain difficult to transplant within a clinically acceptable timeframe (8, 23). In such cases, the pertinent question becomes whether the unacceptable-antigen assignment can be safely refined (risk-adapted delisting), or whether intervention-based access strategies should be considered. This defines the transition from allocation-based barrier reduction to the no-realistic-compatible-option subgroup.

KPD and compatible living-donor pathways represent a distinct compatibility-preserving strategy because they seek a crossmatch-negative donor without pharmacologically crossing an HLA barrier. Integration of compatible pairs can increase chain length, improve match quality, and create additional opportunities for difficult-to-match candidates, but the benefit depends strongly on pool size, donor blood group, donor HLA phenotype, and the candidate’s cPRA/vPRA profile (3).

In candidates with cPRA or vPRA above 95%, KPD alone may be insufficient when the available donor pool is small or when the candidate has broad class II reactivity or rare compatibility requirements. In such settings, combined strategies may be considered, including KPD plus limited low-risk desensitization, KPD plus low-risk ABO-incompatible transplantation, or selection of the least immunologically hazardous donor within a paired-donation chain. Thus, AM allocation and KPD are complementary rather than interchangeable: AM is primarily a deceased-donor allocation-priority strategy that depends on an adequately large organ-sharing network, whereas KPD is a living-donor strategy whose effectiveness depends on registry scale, inclusion of compatible pairs, chain logistics, and the immunologic distribution of enrolled pairs (3, 25).

## 8 When allocation is not sufficient: identifying the no-realistic-compatible-option subgroup

The subgroup requiring escalation is not defined by high sensitization alone, but by the failure of compatible access despite appropriate allocation opportunities. High cPRA, vPRA, or cRF, particularly when combined with low donor frequency or poor matchability, identifies candidates at risk, but the decision to escalate should depend on whether compatible transplantation remains realistic within the relevant allocation system and within the patient’s individual clinical situation (22–24). This distinction is important because allocation policies can substantially change the meaning of a given sensitization level.

Operationally, the no-realistic-compatible-option subgroup should include candidates with persistently poor access despite optimized unacceptable-antigen assignment, standard allocation priority, AM allocation where available, kidney paired exchange, or compatible living-donor pathways where feasible. In candidates who have had sufficient exposure to allocation opportunities, repeated absence of suitable offers, low donor frequency, poor matchability, rare HLA phenotype, and rising waitlist attrition risk should increase concern that compatible transplantation is becoming unrealistic within a clinically meaningful timeframe (26, 27).

Before proceeding to pharmacologic desensitization, centers should also reassess whether all listed unacceptable antigens remain justified. Risk-adapted delisting of selected unacceptable antigens may expand the virtual compatible donor space when antibody evidence is weak, historical, non-complement-binding, or clinically uncertain. This strategy is distinct from antibody removal and should be understood as a structured risk-benefit reassessment of unacceptable-antigen assignment rather than as conventional desensitization (43).

This framework also prevents premature escalation. Candidates with high calculated sensitization but preserved donor frequency, reasonable matchability, or realistic access through AM allocation or paired exchange may still be better served by continued compatible-allocation strategies (3, 5). Conversely, candidates who remain poorly transplantable despite these options represent the population in whom conventional desensitization, risk-adapted delisting, imlifidase, or future immune-cell-targeting strategies become clinically justifiable (2, 10).

A second group requiring proactive consideration consists of sensitized candidates with high clinical urgency on the waiting list. This may include patients with recurrent or impending loss of vascular access, failure of peritoneal dialysis, or other severe dialysis-associated complications. In such patients, waiting longer to document poor access may itself be clinically harmful, and the threshold for timely transplantation through compatible living donation, deceased-donor allocation, or carefully governed access-enabling strategies may be lower. Similarly, candidates with an incompatible living donor and a low probability of successful kidney paired exchange may require individualized evaluation for alternative strategies, particularly when dialysis-related morbidity is high.

## 9 Conventional desensitization: current options and their limitations

Conventional desensitization strategies aim to reduce circulating HLA antibodies, particularly donor-specific antibodies, to a level that permits transplantation across an otherwise prohibitive HLA barrier. In contrast to allocation-based strategies, desensitization does not primarily increase the probability of finding a compatible donor; rather, it attempts to modify the recipient’s antibody profile so that transplantation across an immunological barrier becomes clinically acceptable. This usually requires weeks to months of treatment, repeated antibody monitoring, and highly individualized interpretation of whether previously unacceptable antigens can be safely reconsidered.

The classic intravenous immunoglobulin (IVIG)- and rituximab-based literature demonstrated that transplantation of highly HLA-sensitized candidates can be enabled, particularly in planned or controlled settings such as living-donor transplantation (44, 45). Bortezomib-based protocols added a plasma-cell-directed component to this concept, but the evidence remained heterogeneous and limited by small cohorts, variable protocols, and non-standardized endpoints (46). Thus, conventional desensitization has its clearest role in situations where treatment can be scheduled, antibody kinetics can be monitored iteratively, and the transplant team can define in advance which immunologic risks are acceptable.

The limitations of conventional desensitization are now well recognized. These approaches mainly reduce circulating antibody levels, but they do not reliably eliminate the memory B-cell and plasma-cell compartments that sustain sensitization and can drive early antibody rebound. This explains incomplete efficacy, frequent need for adjunctive or repeated treatment, and the continued risk of antibody-mediated rejection after transplantation (2, 46). HLA-incompatible transplantation after desensitization may provide a survival benefit compared with remaining on the waiting list in selected settings, particularly where waiting-list mortality is high and compatible access is very unlikely (25, 47). However, this benefit must be interpreted in the context of the local allocation system, dialysis outcomes, patient comorbidity, and the immunologic risk accepted at transplantation. For quantitative context, a multicenter study of HLA-incompatible living-donor kidney transplantation reported higher patient survival after HLA-incompatible transplantation than in matched controls who remained on the waiting list or received a deceased-donor transplant, and then in waitlist-only controls, including at 8 years (76.5% vs. 62.9% and 43.9%, respectively) (25).

This distinction is important. Compared with compatible transplantation, desensitization-based HLA-incompatible transplantation generally accepts a higher immunologic risk and requires more intensive treatment, closer monitoring, and readiness to diagnose and treat AMR early (48, 49). In allocation systems where compatible access can be improved through prioritization, kidney paired exchange, or AM allocation, a compatibility-first approach may achieve favorable outcomes for many sensitized candidates without therapeutic barrier crossing (5–7). Therefore, the decision to desensitize should not be based on antibody burden alone, but on whether the expected benefit of earlier transplantation outweighs the risks of AMR, infection, treatment toxicity, cost, and premature graft loss.

More recent pharmacologic strategies have attempted to overcome some of these limitations, but none has yet established a standardized desensitization pathway. Tocilizumab monotherapy had only marginal effects on HLA alloantibodies, whereas add-on interleukin-6 (IL-6) blockade has shown mixed or limited efficacy (50, 51). Clazakizumab generated access-enabling proof-of-concept signals, but confirmation in controlled studies is still required (52). CD38 targeting is mechanistically attractive because it addresses plasma-cell biology; however, isatuximab monotherapy produced only partial desensitization activity and limited effects on cPRA, while daratumumab-based desensitization has so far produced early and not yet practice-changing signals (53, 54). Obinutuzumab demonstrated that deeper CD20-targeted B-cell depletion than with rituximab is feasible, but its routine role in kidney-transplant desensitization remains undefined (55, 56).

Taken together, conventional desensitization should be viewed as a selective escalation strategy rather than a default alternative to compatible allocation. The key clinical question is not merely whether antibody levels can be lowered, but whether the intervention meaningfully changes transplant access, durability of humoral control, and patient-level benefit enough to justify its burden. In candidates with no realistic compatible option, desensitization may be appropriate; in candidates with preserved access through allocation-based compatible pathways, continued pursuit of compatible transplantation will often remain preferable.

## 10 Imlifidase: an access-enabling strategy

Imlifidase introduced a distinct access-enabling concept in highly sensitized kidney transplantation. In contrast to conventional desensitization, which attempts to gradually reduce HLA antibody levels over weeks or months, imlifidase rapidly cleaves circulating IgG within hours and can thereby create a short peri-transplant window in which transplantation across an otherwise prohibitive humoral barrier may become feasible (10). This mechanism is unique because it acts immediately before transplantation rather than by reshaping humoral immunity while the patient is waiting. Consequently, imlifidase-enabled transplantation requires detailed pre-offer HLA antibody characterization, predefined crossmatch logic, and multidisciplinary decision-making before an organ offer is accepted. Because the effect is transient and does not erase humoral memory, the intervention shifts risk from pretransplant incompatibility to early posttransplant rebound management rather than eliminating the underlying alloimmune problem.

Early clinical studies demonstrated that positive crossmatches could be converted after imlifidase, and the international phase 2 trial confirmed rapid crossmatch conversion in highly sensitized recipients (10, 57). Three-year and five-year follow-up studies showed that transplantation can be achieved with meaningful medium-term outcomes in patients who otherwise had extremely limited access to a suitable organ (58–60). Additional single-center five-year data support favorable medium-term outcomes, while emphasizing the importance of DSA rebound monitoring (61). However, the principal biological limitation remains early donor-specific antibody rebound. AMR occurs in a substantial proportion of recipients, around 40% in published cohorts, and this risk must be weighed against the counterfactual probability of receiving a compatible transplant without imlifidase (57, 58). This uncertainty is particularly relevant because available follow-up remains limited for defining durable long-term graft benefit across heterogeneous healthcare systems.

The regulatory and clinical positioning of imlifidase therefore requires precision. According to the European Medicines Agency, imlifidase is indicated for desensitization treatment of highly sensitized adult kidney transplant candidates with a positive crossmatch against an available deceased donor (62). In practice, implementation differs between national and allocation-system frameworks. Some protocols rely on virtual crossmatch-based organ-offer logic followed by post-imlifidase confirmation of crossmatch conversion, whereas others emphasize physical crossmatch conversion before transplantation. Published French, Belgian, Spanish, and international Delphi guidance, together with the German expert consensus report, consistently restrict imlifidase to highly selected candidates who are unlikely to be transplanted through existing allocation and prioritization pathways (11–13, 79). Spanish guidance is consistent with this restricted interpretation (63). **Table 4** summarizes these frameworks.

**Table 4:** Practical positioning of imlifidase in highly sensitized kidney transplant candidates.

Framework	Target population/access concept	Key operational elements	Interpretation for this review
European Medicines Agency/product information	Highly sensitized adult kidney transplant candidates with a positive crossmatch against an available deceased donor	Regulatory indication defines the broad target population; implementation of virtual and/or physical crossmatch logic depends on national allocation and laboratory frameworks	Establishes imlifidase as an access-enabling intervention for selected deceased-donor settings, but does not by itself define a universal allocation algorithm
French consensus guidelines	Highly selected candidates with a positive crossmatch to a deceased donor and very limited compatible access	Restrictive candidate selection; emphasis on DSA characterization, perioperative crossmatch logic, and structured posttransplant surveillance	Provides a national model for translating the regulatory indication into operational candidate-selection and monitoring criteria
Belgian consensus within Eurotransplant	Highly sensitized candidates within a Eurotransplant allocation context who are unlikely to receive a compatible transplant through existing pathways	Operational protocol for imlifidase-enabled deceased-donor transplantation, including center expertise, candidate selection, immunologic assessment, and follow-up	Demonstrates that imlifidase use should be embedded in a governed transplant pathway rather than treated as isolated drug administration
International Delphi consensus	HLA-incompatible deceased-donor kidney transplantation in highly sensitized candidates with no realistic compatible option	Expert consensus on candidate selection, perioperative management, crossmatch conversion, immunosuppression, and posttransplant monitoring	Supports a pathway-based implementation model and reinforces the need for multidisciplinary governance
Spanish guidance for highly sensitized candidates with donor-specific anti-HLA antibodies	Highly sensitized patients with donor-specific anti-HLA antibodies in whom transplantation may require individualized risk assessment	Provides national recommendations on immunologic evaluation, donor-specific antibody assessment, and management of high-risk incompatible transplantation	Reinforces that access-enabling strategies must be adapted to national allocation systems, laboratory practice, and center expertise
Clinical trial and follow-up evidence	Crossmatch-positive, highly sensitized recipients treated with imlifidase in early-phase and follow-up cohorts	Demonstrated rapid crossmatch conversion and feasible transplantation, but with early DSA rebound and antibody-mediated rejection as key vulnerabilities	Defines the evidence base: imlifidase can open a short transplantation window, but its benefit depends on careful selection, surveillance, and rejection management

DSA, donor-specific antibody; EMA, European Medicines Agency. Imlifidase should be understood as a selective access-enabling intervention for candidates with a positive crossmatch to an available deceased donor and no realistic compatible option under existing allocation and prioritization pathways. Published and regulatory frameworks differ in their operational criteria, but consistently emphasize restrictive candidate selection, crossmatch conversion, center expertise, and structured posttransplant monitoring. The now published German expert consensus report provides a Germany-specific framework for risk-adapted HLA delisting, imlifidase use, candidate selection, and posttransplant monitoring (79).

Imlifidase should therefore be selectively applied to highly sensitized candidates with no viable compatible option rather than positioned as an alternative to optimized allocation. Standard allocation, AM allocation, kidney paired exchange, compatible living-donor strategies, and risk-adapted reassessment of unacceptable antigens should be considered first whenever feasible. Imlifidase becomes clinically relevant when these routes are unavailable, unsuccessful, or no longer realistic within a clinically acceptable timeframe, particularly in candidates with severe dialysis-associated morbidity or progressive loss of dialysis options.

Off-label use in living-donor transplantation has been reported in exceptional cases, including a multimodal strategy combining imlifidase and daratumumab in a highly immunized, crossmatch-positive, blood group-incompatible living-donor retransplant recipient (64). Such cases illustrate potential logistical advantages of a planned setting, but they should currently be regarded as exceptional and investigational rather than as part of routine imlifidase positioning.

Because early AMR remains the major limitation of imlifidase-enabled transplantation, the long-term success of this strategy depends not only on crossmatch conversion but also on posttransplant surveillance and timely rejection management. Consensus documents therefore emphasize experienced-center use, perioperative coordination, intensive DSA monitoring, biopsy-based assessment when indicated, and readiness to treat early AMR (11–13). CD38-directed therapies have shown promising activity in AMR and may become relevant for improving the feasibility of barrier-crossing strategies, but their optimal role after imlifidase-enabled transplantation remains to be defined prospectively (65–67). Thus, imlifidase is not simply a drug that permits transplantation; it is a highly selective access-enabling strategy whose benefit depends on careful candidate selection, explicit crossmatch logic, and structured posttransplant governance. Given AMR rates approaching 40%, such risk levels cannot be assumed to be broadly acceptable across all transplant programs. Acceptability depends on the candidate’s counterfactual waitlist risk, the absence of realistic compatible alternatives, drug acquisition and reimbursement, laboratory and surgical logistics, availability of early biopsy and DSA monitoring, and capacity to treat AMR immediately.

## 11 Emerging next-generation strategies: CAR-T cells and bispecific antibodies

The next generation of desensitization strategies aims to address a central limitation of conventional approaches: incomplete control of the memory B-cell and plasma-cell compartments that sustain anti-HLA sensitization. This has stimulated interest in cellular and immune-engaging strategies, including CAR-T cells, BCMA-directed T-cell engagers, and related dual-compartment approaches (14, 15, 17). In particular, chimeric HLA antibody receptor (CHAR)-T cells are designed to target HLA-specific B cells and may provide a more antigen-specific concept than broad B-cell or plasma-cell depletion, although this approach remains preclinical or early translational for transplantation (68).

Among cellular approaches, CAR-T therapy has a clear transplant-specific conceptual rationale and emerging proof-of-concept evidence. In autoimmune disease, CD19-CAR-T-cell therapy has provided broader human evidence that deep B-cell depletion can induce sustained immune resetting, although these findings cannot be directly extrapolated to anti-HLA alloimmunity and the transplant setting (69). A 2024 transplant-focused perspective explained why CAR-T therapy may become relevant for sensitized kidney transplant candidates (14). A 2025 case report suggested that anti-CD19 CAR-T-cell therapy can reduce HLA antibodies in a highly sensitized patient (16). Subsequent clinical reports described kidney transplantation after anti-CD19 or dual CD19/BCMA CAR-T-cell-based reduction of anti-HLA antibodies in three highly sensitized candidates (70, 71). These observations remain proof-of-concept and do not yet support routine implementation. Dedicated trials have been registered to test related hypotheses (NCT07350837 and NCT06056102) (73, 74). A recent review of CHAR-T-cell therapy summarizes the rationale that HLA-specific B-cell elimination could theoretically support selective desensitization or AMR treatment while sparing unrelated B-cell specificities (68).

Another potential treatment option is BCMA-targeting bispecific antibodies, which may also affect the CD19-positive B-cell compartment. A 2026 report on BCMA-targeted T-cell engager therapy combined with B-cell depletion is significant because it demonstrated that plasma-cell-directed immune-engaging therapy may be feasible in refractory HLA sensitization and allowed successful transplantation with prevention of early memory B-cell response and DSA rebound (17). However, universal IgG reduction necessitated IgG substitution, highlighting the limitation of broad and nonspecific plasma-cell depletion. Related organ-transplant and kidney-specific immunotherapy perspectives support the conceptual direction but emphasize that clinical translation remains early and that more clinical data are needed (15, 72). Clinical trials are ongoing to investigate HLA antibody reduction and the posttransplant course after desensitization with BCMA x CD3 bispecific antibodies (NCT05092347 and NCT05106387) (75, 76).

Current data justify inclusion in a forward-looking review, not routine clinical implementation. Future studies are already underway and must clarify durability, infectious toxicity, cost, equitable access, and whether such approaches should be combined with other B-cell-targeting strategies.

## 12 Integrating allocation and intervention: a practical framework

Transplant access for highly sensitized candidates should be managed as a sequential problem rather than as a binary decision for or against desensitization. First, sensitization should be characterized by integrating immunizing history, antibody specificity and plausibility, unacceptable-antigen assignment, SAB interpretation, and virtual or physical crossmatch context (4, 20). Second, transplantability should be assessed within the relevant allocation system using calculated sensitization metrics, donor frequency, favorably matched donor frequency, and matchability where available (18, 19, 28). Third, clinical urgency, dialysis-associated morbidity, comorbidities, and patient preferences should be incorporated into the risk-benefit assessment. Fourth, centers should reassess whether all listed unacceptable antigens remain justified, because selective delisting may be a viable strategy when antibody evidence is weak or when listing a specificity has a disproportionate impact on transplantability. Only after compatible and allocation-based options have been optimized should pharmacologic desensitization or imlifidase-enabled transplantation be considered (3, 5, 9).

Conventional desensitization is a suitable option for planned settings, particularly selected living-donor transplantation, where treatment can be scheduled and immunologic risk can be monitored iteratively (44, 45). Risk-adapted delisting of unacceptables expands the virtual compatible donor pool but requires expert-driven HLA interpretation, crossmatch safeguards, and structured rejection surveillance for early treatment (43). Imlifidase occupies a niche in time-critical deceased-donor settings where a positive crossmatch would otherwise preclude transplantation and the candidate is unlikely to be transplanted through existing allocation and prioritization pathways or needs an urgent transplant. Patients and physicians should understand that AMR may occur after transplantation and that early detection and treatment must be anticipated (11–13).

Taken together, the practical sequence is not allocation versus intervention, but allocation first with intervention as a fallback option when the expected survival, quality-of-life, or access benefit outweighs the risks of continued waiting and barrier-crossing transplantation. Accordingly, high cPRA, vPRA, or cRF should trigger assessment of donor frequency, matchability, received offers, waiting-list trajectory, and patient-level urgency rather than automatic escalation. Posttransplant monitoring remains integral when access is achieved through delisting, desensitization, or imlifidase (11–13, 22, 23, 28).

## 13 Unresolved questions and future directions

Several key questions remain unresolved. First, despite major progress in HLA antibody assessment and consensus terminology, the interpretation of SPA reactivity and the determination of unacceptable antigens still lack full standardization across centers (35–38). Second, comparative evidence remains limited on when escalation is superior to continued waiting for a compatible transplant, particularly in candidates who fall below conventional but still clinically relevant access thresholds (26, 27). Here, dialysis quality and individual dialysis-associated morbidity also enter the equation. Third, the optimal implementation model for imlifidase remains unresolved. European countries have embedded imlifidase differently within regulatory, allocation, and governance frameworks, and it remains unclear which selection criteria and program structures best balance access, safety, AMR risk, and long-term graft benefit (11–13). Fourth, implementation must account for the size and maturity of the relevant organ-sharing environment. In large national or supranational systems with mature allocation algorithms, broad donor exchange, or established KPD networks, continued pursuit of compatibility may be realistic for a larger proportion of highly sensitized candidates. In smaller countries, isolated programs, or low-donor-volume settings, however, donor scarcity and limited paired-donation pools may make an acceptable compatible match unlikely within a clinically tolerable timeframe. In those contexts, desensitization or other access-enabling strategies may become a more central default option, not because incompatibility is preferable, but because the counterfactual waiting risk differs substantially. Fifth, feasibility and resource use remain underdefined. Expert HLA interpretation, repeated antibody testing, KPD infrastructure, imlifidase procurement, perioperative coordination, DSA monitoring, protocol or indication biopsies, AMR treatment readiness, and registry-quality outcome capture all require personnel, laboratory capacity, and funding. The compatibility-first framework is therefore not a purely immunological algorithm; it also depends on whether the healthcare system can provide the infrastructure required to deliver each strategy safely and equitably.

A second major frontier concerns the endpoints used to evaluate desensitization and access-enabling strategies. Reduction in antibody signal, MFI, cPRA, crossmatch reactivity, or donor-specific antibody burden is insufficient as a stand-alone endpoint. Future studies should prioritize clinically meaningful outcomes, including successful transplantation, waitlist attrition, patient survival, graft survival, antibody rebound, rejection, infectious toxicity, quality of life, and cost-effectiveness. These endpoints should be evaluated within allocation-system-specific frameworks, because the clinical value of desensitization depends not only on antibody reduction but also on the counterfactual likelihood of compatible transplantation without intervention and on survival and quality of life on the waitlist.

Finally, emerging immune-cell-targeting strategies raise scientific and governance questions. CD19- and/or BCMA-directed strategies, using CAR-T-cell- or T-cell engager-based regimens, may eventually reshape desensitization (16, 17). Recent KDIGO conference conclusions on B-cell targeting in immune-mediated kidney diseases reinforce that deeper B-cell and plasma-cell targeting requires disease-specific validation, biomarker-guided patient selection, standardized monitoring, and explicit safety frameworks (77). However, key issues of safety, infectious toxicity, durability, cost, and equitable access remain unresolved. A recent German multicentre survey of high-titer ABO-incompatible living-donor kidney transplantation illustrates this implementation problem by documenting wide center-level variation in titer thresholds, escalation pathways, and re-attempt policies in a setting with limited exchange alternatives (78).

## 14 Conclusion

Management of highly sensitized kidney transplant candidates should integrate sensitization burden, unacceptable-antigen assignment, donor-pool access, allocation design, clinical urgency, patient preferences, and expected outcome on dialysis (1, 4, 20). The strongest current evidence supports a sequential strategy in which compatible transplantation remains the goal, whenever feasible. This should include standard allocation, sensitization-aware prioritization, AM allocation, KPD, and compatible living-donor pathways before therapeutic barrier-crossing is considered (3, 5, 9).

For allocation organizations sensitization-related access barriers are modifiable; for transplant centers, the central task is to distinguish candidates who are difficult to transplant from those with no realistic compatible option by integrating donor frequency, matchability, immunizing history, unacceptable-antigen assignment, waiting-list trajectory, and center-specific expertise (18, 19, 22, 24, 28).

In candidates without a realistic compatible option, Conventional desensitization, risk-adapted delisting and imlifidase-enabled transplantation may be justified as selective access-enabling approaches whereas imlifidase use should remain embedded in regulatory, consensus-based, and center-specific governance pathways, and CAR-T-cell and BCMA-directed strategies remain promising but investigational (2, 11–17, 43).

Ultimately, the goal is not merely to enable transplantation, but to achieve durable, safe, and clinically beneficial transplantation. The practical application of this strategy must remain context-sensitive, particularly in smaller or resource-constrained organ-sharing systems where compatible access cannot be assumed.
